# Alemtuzumab-Induced Autoimmune Thyroid Dysfunction

**DOI:** 10.7759/cureus.22751

**Published:** 2022-03-01

**Authors:** Sharanniyan Ragavan, Omar Elhelw, Waseem Majeed, Angelos Kyriacou, Akheel Syed

**Affiliations:** 1 Medical School, University of Manchester, Manchester, GBR; 2 Endocrinology, Salford Royal National Health Service Foundation Trust, Manchester, GBR

**Keywords:** graves’ disease, autoimmune disease, immune reconstitution syndrome, relapsing-remitting multiple sclerosis, thyroid pathology

## Abstract

Alemtuzumab, a humanized monoclonal antibody used as a disease-modifying treatment in relapsing-remitting multiple sclerosis (RRMS), frequently causes autoimmunity as its principal adverse effect. We describe a typical case of a young man treated with two courses of alemtuzumab presenting 18 months later with initial hyperthyroidism due to Graves’ disease (GD) followed by persistent hypothyroidism. We discuss the pathophysiological role of stimulating and blocking thyrotropin receptor antibodies in the development of alemtuzumab-induced autoimmune thyroid dysfunction and clinical challenges posed by spontaneous, bidirectional switching between hyperthyroidism and hypothyroidism. Guidelines recommend monitoring thyroid function pre-treatment and every three months for four years following alemtuzumab treatment. Patient education is crucial for maintaining adherence to monitoring programs.

## Introduction

Alemtuzumab is a humanized monoclonal antibody widely used as a disease-modifying treatment in relapsing-remitting multiple sclerosis (RRMS). Intriguingly, alemtuzumab therapy can lead to predominantly antibody-mediated secondary autoimmune disorders as its adverse effect, including idiopathic thrombocytopenic purpura, anti-glomerular basement membrane disease, neutropenia, hemolytic anemia, vitiligo, and autoimmune thyroid disease (AITD). Here, we present a case of alemtuzumab-induced AITD in a young male previously treated with alemtuzumab for RRMS.

This article was previously presented as a meeting abstract at the EndoBridge 2021 virtual conference (Antalya, Turkey).

## Case presentation

**Figure 1:**
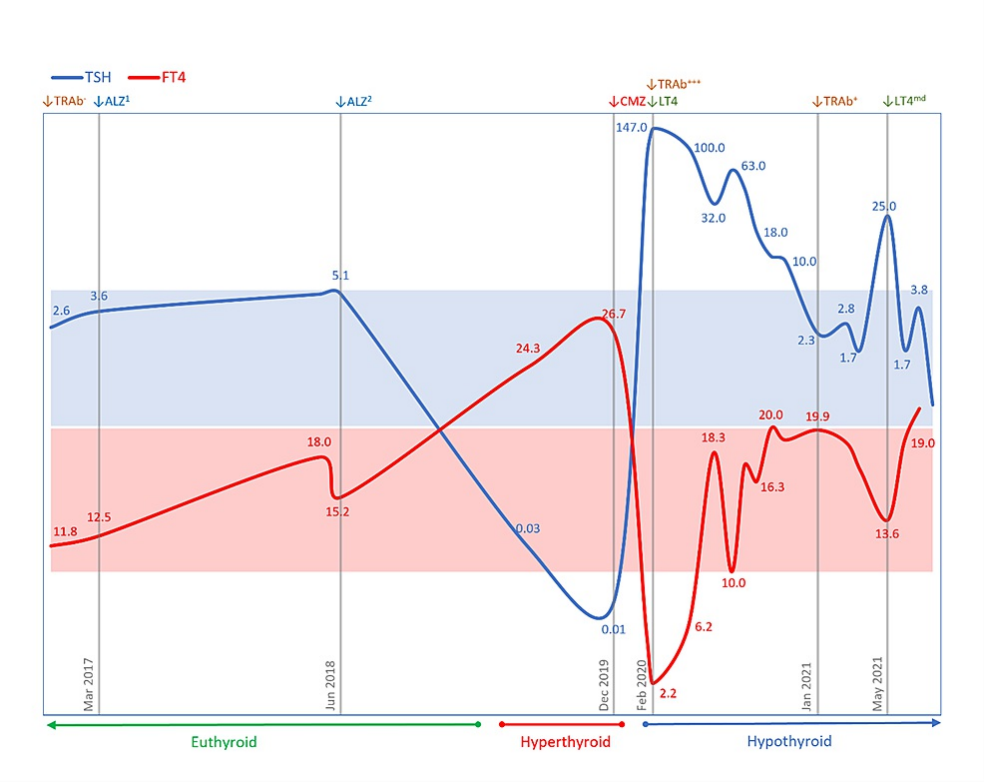
Clinical time-course of alemtuzumab-induced autoimmune thyroid dysfunction Serum thyroid-stimulating hormone (TSH) concentrations depicted by blue trace and TSH reference range (0.35–5.50 mU/L) by light blue stripe. Serum free thyroxine (fT4) concentrations depicted by red trace and fT4 reference range (10.0–20.0 pmol/L) by light red stripe. Timepoint markers (gray vertical droplines) depict the following: TRAb-, negative TSH receptor antibody (TRAb) titer at baseline; ALZ1, alemtuzumab first course; ALZ2, alemtuzumab second course; CMZ, carbimazole commenced; TRAb+++, strongly positive TRAb titer; and LT4, levothyroxine commenced with discontinuation of carbimazole. TRAb+, TRAb remains positive but has reduced in titer; LT4md, missed doses of levothyroxine due to delayed prescription for a week resulted in the rise in TSH (and fall in fT4), indicating persistence of hypothyroidism and continued requirement for levothyroxine to date.

A 27-year-old male was referred to the endocrinology clinic with a six-month history of weight loss without other symptoms. He had a four-year history of relapsing-remitting multiple sclerosis (RRMS) treated with two courses of alemtuzumab 2½ and 1½ years previously. Thyroid function tests (TFTs) had shown low thyroid-stimulating hormone (TSH) at 0.03 (normal range: 0.35­-5.50) mU/L and elevated free thyroxine (fT4) at 24.3 (normal range: 10.0-20.0) pmol/L (Figure 1). He had a family history of hypothyroidism in his maternal grandmother. He had been commenced on carbimazole 20 mg daily and propranolol 40 mg daily for thyrotoxicosis by his neurologist. When seen in the endocrinology clinic six weeks later, physical examination revealed a slow, regular pulse of 41 beats per minute; there were no signs of goiter or peripheral stigmata of Graves’ disease (GD) such as thyroid eye disease, thyroid dermopathy (pretibial myxoedema), or thyroid acropachy. TFTs now showed significantly elevated TSH at 147.0 mU/L and low fT4 at 2.2 pmol/L. TSH receptor antibody (TRAb) titer was 25.6 (normal range: 0.0-0.4) U/L; it had been undetectable at the time of the initial diagnosis of multiple sclerosis four years previously. Thyroid peroxidase antibody (TPOAb) titer was 234 (normal range: <35) IU/L. Carbimazole and propranolol were stopped, and levothyroxine 50 μg daily was commenced with increasing dose titration subsequently up to 150 μg daily. TRAb titer has reduced to 5.2 U/L, but hypothyroidism requiring continued levothyroxine treatment has persisted over two years of follow-up. A brief period of inadvertent missed doses of levothyroxine confirmed ongoing hypothyroidism. In recent weeks, the levothyroxine dose requirement has gradually declined to 75 μg daily, and it is possible that in time it can be stopped completely as the patient’s endogenous thyroid hormone production recovers with the reduction in TRAb activity.

## Discussion

Alemtuzumab, a humanized monoclonal antibody used as a disease-modifying treatment in RRMS given as two courses 12 months apart, suppresses the neuroinflammatory process and reduces relapse rate and disability progression.

The cell surface binding of alemtuzumab results in the near-total depletion of the cluster of differentiation 52-positive (CD52+) lymphoid cells [1,2]. Its subsequent reconstitution results in the alteration of the T cell subset proportions and their properties. These include an increased representation of regulatory T cells and memory T and B lymphocytes and a reduction in autoreactive T cell clones and T cell migration to the CNS. There is also some evidence to suggest that alemtuzumab therapy may promote the restoration of the blood-brain barrier, which is dysregulated in MS. As such, alemtuzumab suppresses the neuroinflammatory responses in RRMS, thus reducing the potential for relapse, thereby delaying disease progression.

However, alemtuzumab’s high efficacy is accompanied by the development of autoimmunity as its principal adverse effect. The mechanism of the secondary autoimmunity remains unclear; it has been attributed to the breakdown in T cell tolerance to self-antigens during immune reconstitution, which occurs during recovery from alemtuzumab-induced T lymphopenia. AITD is the most common antibody-mediated adverse effect of alemtuzumab, with a 42% likelihood among patients on a six-year follow-up [3]. Alemtuzumab-induced AITD commonly occurs 2½ years after the last drug administration [4]. It begins with lymphocytic infiltration into the thyroid tissue and the production of autoantibodies including TRAb, TPOAb, and thyroglobulin antibody. In one large series, most patients develop GD (72%), a smaller proportion develops Hashimoto’s thyroiditis, and some develop thyroid dysfunction of unknown etiology [5]. In contrast, the prevalence of AITD in the general population is 5% [6]; Hashimoto’s thyroiditis is far more frequent than GD, is seen more commonly in women, and increases in incidence with age.

Alemtuzumab therapy in our patient led to the induction of high titer of TRAbs, which were absent pre-treatment, resulting in initial hyperthyroidism due to GD. The differential diagnosis of Hashimoto’s thyroiditis, a chronic autoimmune disease involving the destruction of thyroid follicles resulting in eventual hypothyroidism, can sometimes present with initial thyrotoxicosis (“hashitoxicosis”) as preformed thyroid hormones are released from the inflamed thyroid. Hashitoxicosis is clinically and radiologically virtually indistinguishable from Graves’ hyperthyroidism, even with radioiodine uptake scan [7]. TPOAb, considered as the best serological marker of Hashimoto’s thyroiditis, is elevated in 95% of patients. However, there is an overlap in thyroid antibody production between GD and Hashimoto’s thyroiditis. TPOAb is commonly seen in both conditions but often at high titers in Hashimoto’s thyroiditis. However, TRAb has pathogenic specificity for GD. Our patient had a TPOAb titer sevenfold greater than normal, whereas the TRAb titer was 64-fold greater, consistent with a diagnosis of GD.

The modern TRAb assay has a sensitivity of over 97% and specificity of over 98% and indisputable superiority over clinical diagnosis [8]. It is effective for differentiating GD from other causes of thyrotoxicosis [9]. TRAbs are functionally heterogeneous; stimulating antibodies activate downstream effects resulting in thyrotoxicosis, blocking antibodies cause hypothyroidism, while neutral antibodies neither stimulate nor inhibit TSH receptor action [10]. All types of TRAbs can coexist in the same individual. The clinical and/or biochemical thyroid function status can usually indicate whether the predominant antibodies in question are of stimulating or inhibiting variety [9]. Intriguingly, alemtuzumab-induced GD exhibits spontaneous switching between hyperthyroidism and hypothyroidism, which can be bidirectional, more commonly than in GD presenting in the general population [11]. This phenomenon probably results from a change in the proportion of stimulating versus blocking antibodies. This phenomenon was evident in our patient who initially had hyperthyroidism followed by prolonged hypothyroidism. In our clinical experience, such switching occurs more frequently and more rapidly than in natural GD. However, longitudinal epidemiological data on clinical and biochemical differences between alemtuzumab-induced GD and natural GD is lacking.

The European Thyroid Association recommends measurement of baseline thyroid function in all patients before immune reconstitution therapies (IRT) including alemtuzumab [12]. The risk of thyroid dysfunction post-IRT is increased in people with background thyroid autoimmunity. Most people who develop thyroid dysfunction, however, do not have preexisting thyroid autoantibodies; therefore, routinely, their measurement pre-IRT is not recommended. Preexisting thyroid dysfunction is not a contraindication to IRT but needs monitoring. However, preexisting active thyroid eye disease and cardiac disease where thyrotoxicosis could pose an immediate risk are contraindications to IRT until they have been adequately treated.

**Figure 2:**
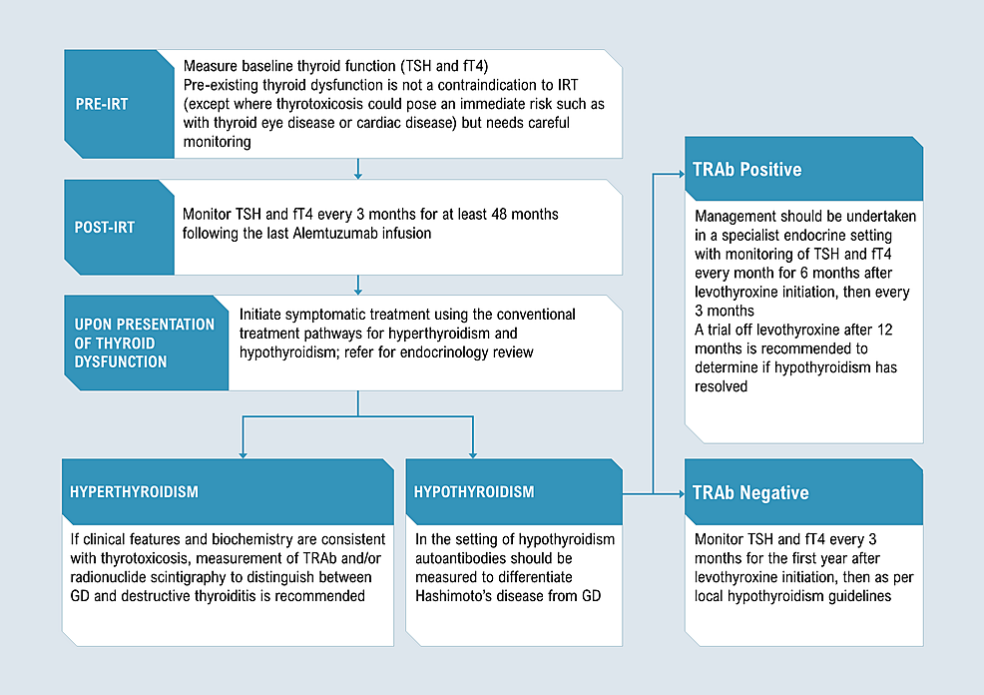
Management of alemtuzumab-induced thyroid dysfunction TSH, thyroid-stimulating hormone; fT4, free thyroxine; IRT, immune reconstitution therapy; TRAb, TSH receptor antibody; GD, Graves’ disease.

Post-IRT, the alemtuzumab product label recommends TFT monitoring every three months for at least 48 months after the last infusion (Figure 2). Routine monitoring of thyroid autoantibodies is not recommended. Once thyroid dysfunction is present, symptomatic treatment should be followed by an early endocrinology review. Management of alemtuzumab-associated AITD relies on conventional treatment pathways for hyperthyroidism and hypothyroidism. In thyrotoxicosis, measurement of TRAb and/or radionuclide scintigraphy to distinguish between GD and destructive thyroiditis is recommended [12]. Ultrasonography can also be considered.

Hyperthyroidism is treated with beta-blockers (non-cardioselective agents such as propranolol are preferred) for symptom control until antithyroid drug therapy with carbimazole/methimazole or propylthiouracil takes effect. Due to the risk of spontaneous switching between hyperthyroidism and hypothyroidism in alemtuzumab-induced GD, block-and-replace therapy with simultaneous antithyroid drug and levothyroxine has found increasing favor [5,12], particularly in the presence of thyroid eye disease, which has a 14% incidence [13]. Consideration of definitive treatment with radioiodine or thyroidectomy is recommended in cases of relapsing GD, drug intolerability, patient preference, or uncontrolled fluctuating disease course [12].

In patients who develop hypothyroidism post-IRT, measurement of both TPOAb (high titers suggestive of Hashimoto’s thyroiditis) and TRAb (diagnostic of GD) is recommended [12]. If TRAbs are absent, monitoring TFTs every three months for the first year after levothyroxine initiation is recommended, then as per local hypothyroidism guidelines. In TRAb-positive patients, specialist monitoring with TFTs every month for six months after levothyroxine initiation and then every three months is endorsed. A trial off levothyroxine after 12 months is suggested to determine if hypothyroidism post-IRT has resolved, especially in patients with positive TRAbs, hypothyroidism preceded by thyrotoxicosis, or a daily levothyroxine dose of up to 50 μg. However, levothyroxine should be continued in women seeking pregnancy, postponing the trial off levothyroxine until after delivery and breastfeeding.

Patient education about monitoring extending for years beyond the administration of alemtuzumab is crucial for the early detection and management of thyroid dysfunction, thus ultimately improving quality of life.

## Conclusions

Alemtuzumab, an effective disease-modifying drug therapy for relapsing-remitting multiple sclerosis, frequently causes autoimmune thyroid dysfunction in a significant proportion of patients. Graves’ disease and Hashimoto’s thyroiditis can have similar clinical presentations. In alemtuzumab-induced autoimmune thyroid dysfunction, additional challenges are posed by spontaneous, bidirectional switching between hyperthyroidism and hypothyroidism. Guidelines recommend monitoring thyroid function pre-treatment and every three months for four years post-treatment. Patient education is crucial for maintaining adherence to monitoring programs. Once thyroid dysfunction is present, systematic treatment with specialist endocrinology guidance should be initiated.
